# Comparative evaluation of shear wave elastography elasticity values in thyroid nodules with cytology results and TI-RADS scoring in differentiation of benign–malignant nodules

**DOI:** 10.1007/s00405-024-08516-0

**Published:** 2024-03-10

**Authors:** Zafer Polat, Muzaffer Elmalı, Asli Tanrivermis Sayit, Cihan Kalkan, Murat Danacı, Mehmet Kefeli

**Affiliations:** 1 Faculty of Medicine, Department of Radiology, Ondokuzmayis University, 55139 Atakum, Samsun Turkey; 2 Faculty of Medicine, Department of Pathology, Ondokuzmayis University, Samsun, Turkey

**Keywords:** Thyroid nodules, ACR TI-RADS, Shear wave elastography, Virtual touch tissue imaging and quantification, Ultrasonography, Fine needle aspiration biopsy, Cytopathology/histopathology

## Abstract

**Purpose:**

The aim of this prospective study was to investigate the diagnostic performance of shear wave elastography (SWE) in differentiating benign and malignant thyroid nodules and their correlation with the American College of Radiology Thyroid Imaging Reporting and Data System (ACR TI-RADS).

**Methods:**

This prospective study included 370 thyroid nodules in 308 patients aged 18–70 years. All the patients underwent B-mode ultrasound (US), Doppler examination, and SWE and were given an ACR TI-RADS risk score before fine needle aspiration biopsy (FNAB) and/or surgery. The correlation between SWE parameters and ACR TI-RADS categories was investigated statistically and compared with histopathologic results. Additionally, the diagnostic performance of SWE was evaluated to distinguish malignant and benign thyroid nodules.

**Results:**

One hundred and thirty-five of the 370 thyroid nodules were malignant, and 235 nodules were benign. The mean shear wave velocity (SWV) value of the malignant nodules (3.70 ± 0.98 m/s) was statistically higher than that of the benign nodules (2.70 ± 0.37 m/s). The best cutoff value of the mean SWV for differentiating benign and malignant nodules was found to be 2.94 m/s (sensitivity 90.4%, specificity 89.9%, positive predictive value 81.3%, negative predictive value 94.1%, *p* < 0.001). The average score of the nodules according to the ACR TI-RADS was 3.57 ± 1.83 in benign nodules and 7.38 ± 2.69 in malignant nodules (*p* ≤ 0.001).

**Conclusion:**

This study showed that combining SWE and TI-RADS improves the specificity of TI-RADS alone in differentiating benign and malignant nodules.

## Introduction

Thyroid nodules are the most common endocrine lesions that are easily detected with ultrasonography (US). The estimated prevalence of thyroid nodules ranges from 4% to 7% by palpation and 20–76% by using high-resolution US in the general adult population [1]. Only 1.6–12% of these nodules are malignant [2]. US is the first preferred and most frequently used method in the evaluation of thyroid nodules, with sensitivity and specificity ranging between 52% and 97% and 26.6% and 83%, respectively [3]. Since the sonographic features of benign and malignant nodules can overlap, the number of unnecessary biopsies increases. Fine needle aspiration biopsy (FNAB) is the gold standard method for distinguishing between malignant and benign thyroid nodules. However, it has some limitations, such as being an invasive method, the possibility of false negative rates, and frequently incomplete or uncertain results [4, 5]. Therefore, alternative methods that can be effective in the diagnosis, follow-up, and selection of nodules to be referred to FNAB have been emphasized. Shear wave elastography (SWE) is a type of US elastography that uses shear waves to assess tissue elasticity and display it in a quantitative manner [6]. The 2016 American Society of Clinical Endocrinologists guidelines reported that US elastography is complementary to gray-scale findings, especially in thyroid nodules with unclear US or cytological findings [5].

The Thyroid Imaging Reporting and Data System (TI-RADS) has been developed to provide standardization in the evaluation and reporting of thyroid nodules. The American College of Radiology (ACR) TI-RADS uses a scale of points according to size, composition, echogenicity, shape, margin, calcification, or echogenic foci, which are scored individually. The feature scores are summed to arrive at the final classification of the risk level, which ranges from TR1 (benign) to TR5 (highly suspicious of malignancy) [7, 8].

A few studies in the literature have revealed the diagnostic success of SWE in thyroid nodules in the differentiation of benign and malignant [9]. The main purpose of our study is to compare the elasticity values obtained with the SWE-Virtual Touch Tissue Imaging Quantification (VTIQ) technique in the differentiation of benign and malignant thyroid nodules with cytology results and to investigate the correlation with ACR TI-RADS scoring.

## Materials and methods

### Patients

This prospective study included 440 patients aged 18–70 years with 528 thyroid nodules. They had suspicious features for malignancy during their sonography controls and were referred for thyroid biopsy between June 2019–2020; there was no previous history of treatment. Exclusion criteria were patients who could not adapt to ultrasonographic examination, those with very deep and superficial nodules, nodules with dense eggshell calcification or dense wall macrocalcification, pure cystic nodules, patients who received radiotherapy to the neck region, or patients who had undergone surgical treatment.

### B-mode ultrasonography and shear wave elastography

Gray-scale US and SWE examinations were performed by a radiologist (Z.P.) with experience in thyroid imaging using a Siemens ACUSON S2000 (Siemens Medical Solutions, Mountain View, CA, USA) US device. Gray-scale US examination was performed with 18 and 9 MHz linear transducers, and SWE examination was performed with a 9 MHz linear transducer.

In B-mode US, thyroid nodules were evaluated according to the ACR TI-RADS 2017 guidelines [8]. After the B-mode examination, acoustic radiation force impulse (ARFI) imaging with VTIQ was performed only on nodules that were expected to undergo FNAB. The nodule was included in a region of interest (ROI) elasticity box, and a shear wave velocity (SWV) map was created. In this map, the hard areas of the nodule are coded in red, the soft areas in blue, and the medium areas in green. According to the size of the nodule and the homogeneity of the signal on the SWV map, 5–13 small ROIs of 1.2 × 1.2 mm, sufficient to sample the majority of the nodule, were placed in the nodule where the measurement was made. ROI boxes were avoided from being placed in cystic areas, in macrocalcification foci, and in areas with very low signals. Measurements were repeated twice for intraobserver agreement.

In VTIQ, the shear wave speed is expressed in m/s. The optimal cut-off value of the minimum, maximum, and mean SWE measurements to distinguish between benign and malignant nodules with the highest sensitivity, specificity, and accuracy was determined using receiver operating characteristic (ROC) curve analysis.

### FNAP procedure

A total off 40 patients were included in the study. One nodule in 359 patients, two nodules in 75 patients, three nodules in five patients, and four nodules in one patient were detected. After ultrasonographic examination, FNAB was performed on a more suspected nodule. All patients provided written informed consent before biopsy. FNAB was performed by a radiologist with experience in thyroid gland pathologies (20 years) using a 22–27 gauge needle under US guidance. FNAB results were reported according to the Bethesda system. Patients with non-diagnostic or abnormal FNAB who refused repeated FNAB, refused recommended surgery, or did not return for follow-up were excluded from the study.

### Cytological/histopathological examination

One hundred fifty-eight of the 528 thyroid nodules were excluded from the study. Sixty-two of the 158 nodules were nondiagnostic, 70 were atypia of undetermined significance, 18 were suspected of follicular neoplasia, and 8 were suspected of malignancy. Benign or malignant pathology results were obtained with FNAB and/or surgical specimens in a total of 308 patients. As a result, statistical analysis was performed on 370 nodules in 308 patients**.**

**Fig. 1:**
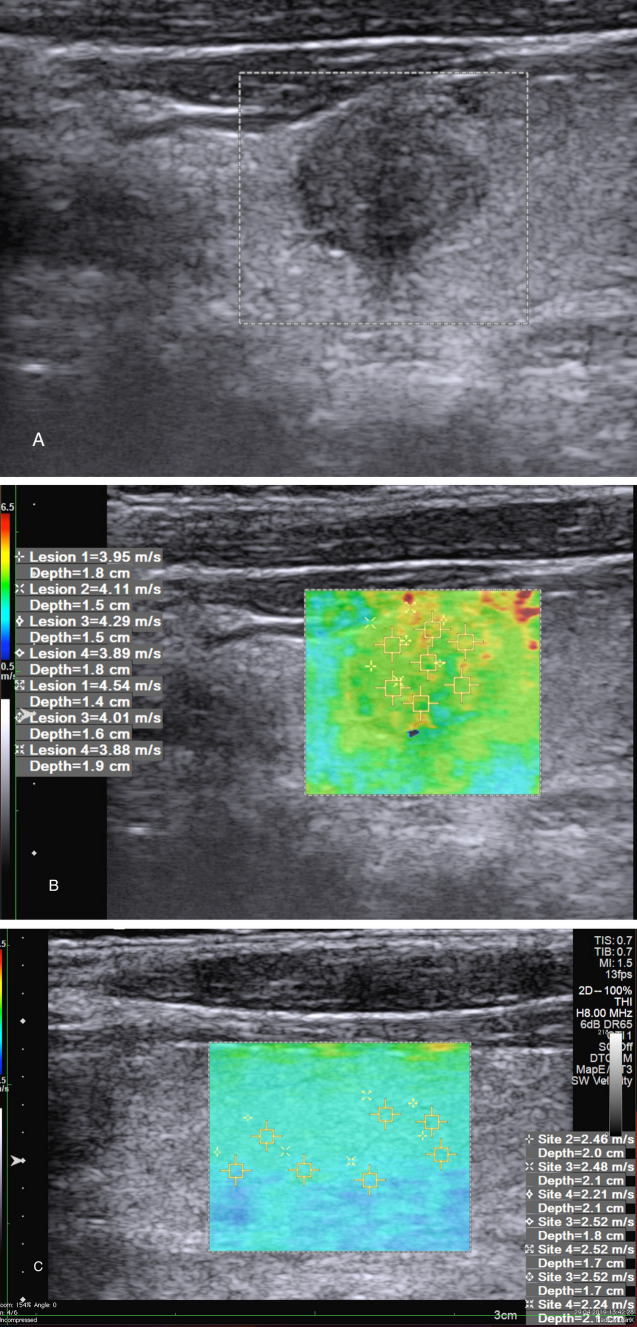
A 58 year old female patient with surgically proven papillary carcinoma. **A**, B-mode ultrasound shows a hypoechoic solid nodule with slightly irregular margin in the left thyroid gland, upper pole. **B**, Shear wave image with thyroid nodule. SWV measurements of nodule was as 4.09 m/s and **C**, for thyroid tissue was 2.42 m/s

Two hundred and thirty-five nodules were found to be benign (63.5%), and 135 were malignant (36.5%). While 212 of the benign nodules were diagnosed with FNAB, histopathological diagnosis was obtained in 30 nodules. Forty-one of the 135 malignant nodules were diagnosed cytologically only with FNAB, and 94 of the malignant nodules were diagnosed histopathologically (Fig. 1).

### Statistical analysis

Statistical analyses were performed using SPSS statistical software, version 15 (SPSS Inc., Chicago, IL, USA). The Kolmogorov–Smirnov test was used to determine whether continuous variables were normally distributed. The *p*-value of the test above 0.05 was considered to conform to the normal distribution. The Pearson chi-square test was used to compare categorical variables. In the comparison of the two groups of independent variables, the Student’s *t*-test was used for normally distributed continuous variables, and the Mann–Whitney *U*-test was used for non-normally distributed continuous variables. The Kruskal–Wallis test was used to compare variables with more than two independent groups. Descriptive analyses results were expressed as the mean and standard deviations for normally distributed variables. The median (minimum–maximum values) was used for non-normally distributed variables. Categorical variables were expressed as percentages. A value of *p* < 0.05 was considered significant in all statistical analyses. ROC curve analysis was used to determine a cutoff value for the differentiation of malignant nodules and to calculate sensitivity and specificity levels.

## Results

### Patients

Sixty-four (20.8%) of the 308 patients were male, and 244 (79.2%) were female. The mean age of all patients was 50.60 ± 13.37. The mean age of the patients with malignant thyroid nodules was significantly lower than that of those with benign nodules (47.04 ± 13.74 vs. 52.45 ± 12.82, respectively, p = 0.001). Of the 235 benign nodules, 48 (65.3%) were detected in male patients and 187 (63.8%) were detected in female patients. Of the 135 malignant nodules, 29 (37.7%) were detected in male patients and 106 (36.2%) were detected in female patients.

### B-mode ultrasonography

The mean maximum size measured by US in benign nodules was 20.14 ± 8.95 mm, while it was 16.83 ± 9.45 mm in malignant nodules (*p* < 0.001). There was no nodule with extrathyroidal extension. The B-mode US findings are shown in Table 1.

**Table 1:** Comparison of malignant and benign thyroid nodules according to B-mode ultrasound findings

Variables	Thyroid nodules	Benign (*n* = 235)	Malignant (*n* = 135)	*p*
Size (mm)				
Mean ± SD	18.96 ± 9.25	20.14 ± 8.95	16.83 ± 9.45	** < 0.001**
Median (min–max)	17 (4–58)	19 (4–58)	14 (4–51)	
Nodule localization				
Right	182 (49.2%)	113 (62.1%)	69 (37.9%)	0.118
Left	176 (47.6%)	111 (63.1%)	65 (36.9%)	
İsthmus	12 (3.2%)	11 (91.75)	1 (8.3%)	
Internal sturucture				
Solid	245 (66.2%)	126 (51.4%)	119(48.6%)	** < 0.001**
Semisolid	111 (30%)	95 (85.6%)	16 (14.4%)	
Spongiform	14 (3.8%)	14 (100%)	0	
Echogenicity				
Prominent hypoechogenicity	20 (5.4%)	3 (15%)	17 (85%)	** < 0.001**
Hypoechogenicity	204 (55.1%)	115 (48.9%)	89 (43.6%)	
Isoechogenicity	109 (29.5%)	89 (81.7%)	20 (18.3%)	
Hyperechogenicity	37 (10%)	28 (75.7%)	9 (24.3%)	
Nodul contours				
Well-defined	264 (71.4%)	207 (78.4%)	57 (21.6%)	** < 0.001**
Lobule–microlobule	97 (26.2%)	21 (21.6%)	76 (78.4%)	
Ill-defined	9 (2.4%)	7 (77.4%)	2 (22.6%)	

### TI-RADS findings

The scoring results of the TI-RADS examination are shown in Table 2. In the statistical analysis, the median TI-RADS score of malignant nodules was found to be significantly higher than that of benign nodules (*p* < 0.001). In the statistical analysis, it was determined that the probability of detecting malignancy increased gradually from Category 1 to 5 (*p* < 0.001).

**Table 2:** Comparison of malignant and benign nodules according to the results of TI-RADS score

Variables	Thyoid nodules	Benign (*n* = 235)	Malignant (*n* = 135)	*p*
Total score				
Mean ± SD	4.88 ± 2.74	3.57 ± 1.83	7.38 ± 2.69	** < 0.001**
Median (min–max)	4 (0–15)	3 (0–11)	7 (2–15)	
TI-RADS category				
1	14 (3.8%)	14 (100.0%)	0	** < 0.001**
2	34 (9.2%)	32 (94.1%)	2 (5.9%)	
3	112 (33.3%)	103 (92.0%)	9 (8.0%)	
4	107 (28.9%)	69 (64.5%)	38 (35.5%)	
5	103 (27.8%)	17 (16.5%)	86 (83.5%)	

*TI-RADS* thyroid ımaging reporting and data system

ROC curve analysis was used to examine the validity of the total score obtained as a result of the TI-RADS examination in the differentiation of malignant nodules. Based on the ROC curve TI-RADS score predicting malignancy, a cut-off of 4.5 area under crve (AUC) = 0.877, *p* ≤ 0.001) has a sensitivity and specificity of 81.5% and 78.7%, respectively; the accuracy rate was 79.7%, the positive predictive value (PPV) was 69%, and the negative predictive value (NPV) was 88.1%.

### Shear wave elastography findings

The SWE measurement results are presented in Table 3. In the statistical analysis, a significant difference was found between malignant and benign nodules in terms of SWV_min_, SWV_max_, SWV_median_, SWV_mean_, and SWV_ratio_ values (for all variables, *p* < 0.001).

**Table 3:** Comparison of malignant and benign thyroid nodules according to shear-wave elastography values

Variables	Total nodules (*n* = 370)	Benign (*n* = 235)	Malignant (*n* = 135)	*p*
SWV_min_				
Mean ± SD	2.75 ± 0.63	2.47 ± 0.35	3.24 ± 0.71	** < 0.001**
Median (min–max)	2.59 (1.90–5.92)	2.40 (1.90–4.49)	3.04 (2.08–5.92)	
SWV_max_				
Mean ± SD	3.43 ± 1.13	2.96 ± 0.44	4.25 ± 1.44	** < 0.001**
Median (min–max)	3.02 (2.03–9.09)	2.88 (2.03–8.71)	3.76 (2.52–9.09)	
SWV_median_				
Mean ± SD	3.05 ± 0.81	2.69 ± 0.38	3.67 ± 0.98	** < 0.001**
Median (min–max)	2.76 (2.10–7.88)	2.62 (2.10–4.92)	3.36 (2.18–7.88)	
SWV_mean_				
Mean ± SD	3.06 ± 0.82	2.7 ± 0.37	3.70 ± 0.98	** < 0.001**
Median (min–max)	2.72 (2.02–7.23)	2.62 (2.02–4.90)	3.35 (2.33–7.23)	
SWV_ratio_				
Mean ± SD	1.28 ± 0.36	1.13 ± 0.22	1.55 ± 0.44	** < 0.001**
Median (min–max)	1.16 (0.92–3.28)	1.09 (0.92–2.21)	1.41 (0.96–3.28)	
SWV_mean-parenchyma_				
Mean ± SD	2.39 ± 1.76	2.39 ± 0.16	2.39 ± 0.20	0.635
Median (min–max)	2.38 (1.94–3.39)	2.38 (1.94–2.89)	2.38 (2.05–3.39)	

*SWV* shear wave velocity; *SD* standard deviation

The SWV ratio cut-off value of greater than 1.22 m/s yielded sensitivity and specificity values of 88.9% and 85.5% (AUC = 0.893, *p* ≤ 0.001), respectively. The accuracy rate was 86.7%, the PPV was 77.9%, and the NPV was 93.1% for the diagnosis of malignant nodules.

Comparison of SWE measurement results with TI-RADS results are shown in Tables 4 and 5. When ACR TI-RADS and SWE were used together, 14 (12.5%) of 112 nodules, identified as Category 3, were transferred to Category 4 because the SWV_mean_ cut-off value was ≥ 2.94. Sixty-two (57.9%) of 107 nodules, identified as Category 4, were transferred into Category 3 due to their SWV_mean_ value < 2.94 m/s. Of the 14 nodules that transferred to Category 4 out of Category 3 nodules, seven were malignant and seven were benign. Of the 62 nodules that were transferred from Category 4 to Category 3, six were malignant and 56 were benign (Table 6). In the validity analysis of this method, the sensitivity and specificity were 92.6% and 84.25%, respectively, for distinguishing thyroid nodules from benign to malignant. In addition, the PPV was 77%, and the NPV was 95%.

**Table 4:** Comparison of SWE measurement results with TI-RADS scores

Variables	TI-RADS 1 (*n* = 14)	TI-RADS 2 (*n* = 34)	TI-RADS 3 (*n* = 112)	TI-RADS 4 (*n* = 107)	TI-RADS 5 (*n* = 103)	*p*
SWV_min_						
Mean ± SD	2.35 ± 0.2	2.47 ± 0.25	2.47 ± 0.33	2.65 ± 0.45	3.30 ± 0.81	** < 0.001**
Median (min–max)	2.34 (2.07–2.64)	2.47 (1.90–3.15)	2.42 (1.90–4.12)	2.58 (1.93–4.56)	3.08 (1.93–5.92)	
SWV_max_						
Mean ± SD	2.84 ± 1.58	2.93 ± 0.28	2.97 ± 0.47	3.20 ± 0.62	4.41 ± 1.60	** < 0.001**
Median (min–max)	2.87 (2.55–3.13)	2.91 (2.28–4.41)	2.88 (2.23–5.75)	3.01 (2.03–5.84)	3.87 (2.33–9.09)	
SWV_mean_						
Mean ± SD	2.57 ± 1.45	2.69 ± 0.24	2.70 ± 0.36	2.91 ± 0.50	3.80 ± 1.09	** < 0.001**
Median (min–max)	2.59 (2.30–2.81)	2.6 (2.24–3.48)	2.63 (2.15–4.90)	2.79 (2.02–5.21)	3.46 (2.15–7.23)	
SWV_median_						
Mean ± SD	2.56 ± 0.18	2.68 ± 0.27	2.69 ± 0.36	2.91 ± 0.51	3.76 ± 1.09	** < 0.001**
Median (min–max)	2.61 (2.21–2.82)	2.69 (2.11–3.50)	2.60 (2.19–4.92)	2.77 (2.10–5.00)	3.41 (2.18–7.88)	
SWV_ratio_						
Mean ± SD	1.05 ± 0.06	1.12 ± 0.10	1.12 ± 0.13	1.23 ± 0.21	1.61 ± 0.49	** < 0.001**
Median (min–max)	1.06 (0.96–1.16)	1.11 (0.99–1.54)	1.09 (0.93–1.79)	1.17 (0.92–2.21)	1.45 (0.94–3.28)	

*SWV* shear wave velocity; *SWE* shear wave elastography; *TI-RADS* thyroid ultrasound imaging reporting and data system

**Table 5:** Comparison of SWE measurement results by combined TI-RADS categories

Variables	TI-RADS 1, 2 vs 3 (*n* = 160)	TI-RADS 4, 5 (*n* = 210)	*p*
SWV_min_			
Mean ± SD	2.46 ± 0.30	2.97 ± 0.72	** < 0.001**
Median (min–max)	2.42 (1.90–4.12)	2.83 (1.93–6.47)	
SWV_max_			
Mean ± SD	2.95 ± 0.42	3.79 ± 1.34	** < 0.001**
Median (min–max)	2.88 (2.23–5.75)	3.47 (2.03–9.77)	
SWV_mean_			
Mean ± SD	2.69 ± 0.33	3.35 ± 0.95	** < 0.001**
Median (min–max)	2.62 (2.11–4.90)	3.14 (2.10–7.23)	
SWV_median_			
Mean ± SD	2.68 ± 0.33	3.33 ± 0.95	** < 0.001**
Median (min–max)	2.62 (2.11–4.92)	3.17 (2.10–7.88)	
SWV_ratio_			
Mean ± SD	1.11 ± 0.12	1.42 ± 0.42	** < 0.001**
Median (min–max)	1.09 (0.93–1.79)	1.33 (0.92–3.28)	

*SWV* shear wave velocity; *SWE* shear wave elastography; *TI-RADS* thyroid ultrasound ımaging reporting and data system

**Table 6:** Management of category 3 and 4 nodules according to ACR TI-RADS and comparison of the effectiveness of the SWVmean cut-off value with TI-RADS

ACR TI-RADS 3 nodules	The histopathologic result was benign				The histopathologic result was malignant			
	SWVmean ≤ 2.94 m/s		SWVmean>2.94 m/s		SWVmean ≤ 2.94 m/s		SWVmean>2.94 m/s	
	Number	%*	Number	%*	Number	%**	Number	%**
FNAB recommended	34	14.5	1	0.4	2	1.5	1	0.7
Follow-up recommended	34	14.5	2	0.8	0	0	2	1.5
FNAB and/or follow-up not recommended	28	11.9	4	1.7	0	0	4	3
ACR TI-RADS 4 nodules								
FNAB recommended	40	17	5	2.1	5	3.7	17	12.6
Follow-up recommended	10	4.2	4	1.7	1	0.7	8	5.9
FNAB and/or follow-up not recommended	6	2.5	4	1.7	0	0	7	5.2

*SWV* shear wave velocity; *FNAB* fine needle aspiration biopsy; *TI-RADS* thyroid ultrasound imaging reporting and data system

^*^All benign nodules

^**^All malignant nodules

## Discussion

Benign thyroid lesions are softer than malignant ones. Recently, the stiffness of these tissues or lesions has been measured objectively and quantitatively using the ARFI method with the VTIQ technique. During VTIQ, during short durations (0.03–0.04 ms), acoustic pulses cause small localized tissue displacements. The more elastic tissues displace more than the stiffer tissues. Displacements create shear wave propagation, which can be calculated [9].

SWV is the numerical value (m/s) that distinguishes hard and soft tissues. It has been reported in the literature that ARFI-VTIQ is used in many tissues to differentiate between benign and malignant lesions [9–12]. In addition, ARFI has high sensitivity and specificity in the differential diagnosis of benign–malignant nodules in the thyroid gland [9]. Here, we evaluated the contribution of the SWE VTIQ technique to ACR TI-RADS scoring for the differentiation of malignant thyroid nodules.

Zhang et al. [13] conducted a comprehensive study using ARFI-VTQ in the differential diagnosis of benign thyroid nodules from malignant nodules. They showed that the mean SWV value of malignant nodules was significantly higher than that of benign nodules (6.34 ± 2.58 vs 2.15 ± 0.59 m/s, respectively). In Hou et al.’s [14] study**,** the mean SWV value of malignant nodules was found to be significantly higher than that of benign nodules (3.10 ± 1.08 vs 2.03 ± 0.42 m/s, respectively). In addition, they reported a cut-off value of 2.42 m/s with sensitivity, specificity, and accuracy for differentiating between benign and malignancy lesions of 80.00%, 89.23%, and 87.05%, respectively. In our study, consistent with the literature, the mean SWV value of malignant thyroid nodules was found to be significantly higher than that of benign nodules (3.70 ± 0.98 vs 2.7 ± 0.37 m/s, respectively). The SWV ratio value was significantly higher in malignant nodules than in benign nodules (1.55 ± 0.44 vs 1.13 ± 0.22, respectively). In addition, in the differential diagnosis of benign–malignant nodules, the mean SWV cut-off value was found to be 2.94 m/s, with a sensitivity of 90.4%, a specificity of 88.9%, and an accuracy of 88.1%.

There are studies conducted with TI-RADS scoring in the literature. Xu et al. [15] reported a large-scale study including a total of 2465 thyroid nodules, 1460 of which were benign and 1005 were malignant, in 2031 patients. In this study, the cut-off value for the TI-RADS score was reported as 4 for the differentiation between benign and malignant nodules, with 96% sensitivity and 52% specificity [15].

Hang et al. evaluated 298 thyroid nodules, 177 of which were malignant in 262 patients. The cut-off value of the TI-RADS scoring system was found to be 5 out of a total score in the differentiation of benign and malignant nodules. The sensitivity and specificity for this value were 89.8% and 73.6%, respectively [16].

In our study, the median TI-RADS score of malignant nodules was found to be significantly higher than that of benign nodules (*p* < 0.001). In the statistical analysis, it was determined that the probability of detecting malignancy increased gradually from Category 1 to Category 5 (*p* < 0.001). Here, the cut-off value of the TI-RADS score was found to be 4.5 with 81.5% sensitivity and 78.7% specificity (AUC = 0.877, *p* < 0.001). In addition, in our study, TI-RADS scoring and SWE measurements were performed on the nodules to understand whether the SWE VTIQ technique is complementary and selective in directing thyroid nodules to FNAB and to evaluate the correlation of SWE measurement parameters with ACR TI-RADS.

Additionally, when all SWE values (SWV_min_, SWV_max_, SWV_mean_, SWV_median_, and SWV_ratio_) were compared according to the TI-RADS categories, a significant difference was found between the categories (*p* < 0.001 for each). Furthermore, in our study, 14 (12.5%) of 112 nodules identified as Category 3 were included in Category 4 because their SWV_mean_ cut-off value was ≥ 2.945 m/s. Of the 14 nodules assigned to Category 4 out of Category 3 nodules, seven were malignant and seven were benign. Sixty-two (57.9%) of 107 nodules detected as Category 4 were included in Category 3 because the SWV_mean_ cut-off value was < 2.945 m/s. Of the 62 nodules included in the Category 3 group from Category 4 nodules, six were malignant and 56 were benign. After editing, nodules of Categories 1, 2, and 3 were accepted as benign. Nodules of Categories 4 and 5 were accepted with a high probability of malignancy; 92.6% sensitivity and 84.2% specificity were found for TI-RADS in the differentiation from benign to malignant. There was no significant change in sensitivity with the combined use of SWE and TI-RADS (from 91.8% to 92.6%); however, a significant increase was found in specificity (from 63.4% to 84.2%). We predicted that SWE may make an additional contribution to the decision of FNAB for thyroid nodules, in addition to the ACR TI-TRADS guidelines.

Our study has some limitations. TI-RADS scoring and SWE examinations were performed by the same radiologist, which may have led to selection bias regarding the practitioner. Although intraobserver agreement was checked, interobserver agreement was not evaluated. The main limitations of our study were that the patient group consisted of selected cases for FNAB with suspicious findings in terms of malignancy, and the prevalence of malignant nodules was higher (36.5%) in the cases within the scope of the study since our study was conducted in a tertiary university hospital.

## Conclusion

In conclusion, ARFI-VTIQ has high sensitivity and specificity between benign and malignant nodules, and it is thought that it may contribute positively to the clinical evaluation of thyroid nodules. Our study showed that a combination of SWE and TI-RADS scores increased the specificity of TI-RADS alone in differentiating between benign and malignant thyroid nodules and that it can reduce unnecessary biopsies.

## Abbreviations

ARFI: Acoustic radiation force impulse
ATA: American Thyroid Association
ACRTIRADS: American College of Radiology Thyroid Imaging Reporting and Data System
FNAB: Fine needle aspiration biopsy
NPD: Negative predictive value
PPD: Positive predictive value
ROC: Receiver operating characteristic
ROI: Region of interest
SWE: Shear wave elastography
SWV: Shear wave velocity
TI-RADS: Thyroid ultrasound imaging reporting and data system
US: Ultrasound
VTIQ: Virtual touch tissue imaging quantification
