# Correction: Comparative evaluation of shear wave elastography elasticity values in thyroid nodules with cytology results and TI-RADS scoring in differentiation of benign–malignant nodules

**DOI:** 10.1007/s00405-024-08637-6

**Published:** 2024-04-03

**Authors:** Zafer Polat, Muzaffer Elmalı, Asli Tanrivermis Sayit, Cihan Kalkan, Murat Danacı, Mehmet Kefeli

**Affiliations:** 1 Faculty of Medicine, Department of Radiology, Ondokuzmayis University, 55139 Atakum, Samsun Turkey; 2 Faculty of Medicine, Department of Pathology, Ondokuzmayis University, Samsun, Turkey

ORCID: http://orcid.org/0000-0003-2861-156X

**Correction: European Archives of Oto-Rhino-Laryngology** 10.1007/s00405-024-08516-0

In the original publication of the article, in the section “Materials and methods”, on FNAP procedure, the beginning sentence was published incorrectly as “A total of 40 patients were included in the study”. The correct sentence should read as “A total of 440 patients were included in the study”.

